# A convolutional neural network for fast upsampling of undersampled tomograms in X-ray CT time-series using a representative highly sampled tomogram

**DOI:** 10.1107/S1600577519003448

**Published:** 2019-04-23

**Authors:** Dimitrios Bellos, Mark Basham, Tony Pridmore, Andrew P. French

**Affiliations:** a School of Computer Science, Jubilee Campus, University of Nottingham, Wollaton Road, Nottingham NG8 1BB, UK; b Diamond Light Source Ltd, Harwell Science and Innovation Campus, Didcot, Oxfordshire OX11 0DE, UK

**Keywords:** convolutional neural networks, projection upsampling, sinogram upscaling, time-resolved X-ray computed tomography

## Abstract

X-ray computed tomography and, specifically, *time-resolved* volumetric tomography data collections (4D datasets) routinely produce terabytes of data, which need to be effectively processed after capture. This is often complicated due to the high rate of data collection required to capture at sufficient time-resolution events of interest in a time-series, compelling the researchers to perform data collection with a low number of projections for each tomogram in order to achieve the desired ‘frame rate’. It is common practice to collect a representative tomogram with many projections, after or before the time-critical portion of the experiment without detrimentally affecting the time-series to aid the analysis process. For this paper these highly sampled data are used to aid feature detection in the rapidly collected tomograms by assisting with the upsampling of their projections, which is equivalent to upscaling the θ-axis of the sinograms. In this paper, a super-resolution approach is proposed based on deep learning (termed an upscaling Deep Neural Network, or UDNN) that aims to upscale the sinogram space of individual tomograms in a 4D dataset of a sample. This is done using learned behaviour from a dataset containing a high number of projections, taken of the same sample and occurring at the beginning or the end of the data collection. The prior provided by the highly sampled tomogram allows the application of an upscaling process with better accuracy than existing interpolation techniques. This upscaling process subsequently permits an increase in the quality of the tomogram’s reconstruction, especially in situations that require capture of only a limited number of projections, as is the case in high-frequency time-series capture. The increase in quality can prove very helpful for researchers, as downstream it enables easier segmentation of the tomograms in areas of interest, for example. The method itself comprises a convolutional neural network which through training learns an end-to-end mapping between sinograms with a low and a high number of projections. Since datasets can differ greatly between experiments, this approach specifically develops a lightweight network that can easily and quickly be retrained for different types of samples. As part of the evaluation of our technique, results with different hyperparameter settings are presented, and the method has been tested on both synthetic and real-world data. In addition, accompanying real-world experimental datasets have been released in the form of two 80 GB tomograms depicting a metallic pin that undergoes corruption from a droplet of salt water. Also a new engineering-based phantom dataset has been produced and released, inspired by the experimental datasets.

## Abstract 2

A convolutional neural network has been designed to quickly and accurately upscale the sinograms of X-ray tomograms captured with a low number of projections, effectively increasing the number of projections. This is particularly useful for tomograms that are part of a time-series as, in order to capture fast-occurring temporal events, tomograms have to be collected quickly, requiring a low number of projections. The upscaling process is facilitated using a single tomogram with a high number of projections for training, which is usually captured at the end or the beginning of the time-series when capturing the tomogram quickly is no longer needed.

## 1. Introduction   

Over recent decades, X-ray computed tomography has become more and more popular, allowing researchers to capture the hidden inner structure of many different systems. Applications of computed tomography can be found in many fields, including medicine, biology, material science and so on. However, there are cases where the time of exposure of the imaged sample must be limited, allowing only for a small number of projections to be captured. One of these cases is during the acquisition of *time-resolved* volumetric tomography data collections (4D datasets), where multiple tomograms with smaller numbers of component projections are collected to enable capture of fast-occurring temporal events. However, this comes at a cost. As a lower amount of data is used in the reconstruction process, the imaged sample is not well described in each tomogram, making its reconstruction an ill-posed problem. This results in poor quality reconstructions, where the level of noise is high and ray artefacts are prevalent. One computational way to address this issue is by artificially increasing the number of projections by upscaling the component sinograms of the respective tomograms along their θ-axis (Section 2.2). This approach in turn can be viewed as an application of super-resolution, but within the sinogram space. Super-resolution, and generally the act of increasing the resolution of an image, has been a very active area of research in recent years. The main goal is the recovery of an image with a higher pixel count, that more accurately describes what is pictured, using one or multiple images of low pixel count to achieve this.

Over recent years, super-resolution has been investigated for the task of upscaling images. In the published literature, there are in general four categories of super-resolution algorithms: (i) edge-based methods, (ii) methods using predictive models, (iii) statistical methods and (iv) patch-based (sometimes called example-based) methods, which are where most state-of-the-art algorithms sit. These methods have been thoroughly investigated and evaluated in Yang *et al.*’s work (Yang *et al.*, 2014 ▸). Internal example-based methods exploit the similarity of areas within the same image. In particular, Glasner *et al.* (2009 ▸) used exemplar patches across different scales of the same image, while improved variations were proposed later in which the execution time is reduced (Freedman & Fattal, 2011 ▸), and where the performance has been improved using sparse coding (Yang *et al.*, 2010*a* ▸). External example-based methods work with the aid of an external database, using it to learn a mapping between patches with low and high pixel count. One of the first of these methods was proposed by Freeman *et al.* (2002 ▸), who used pairs of low/high pixel count patches and a nearest neighbour strategy to synthesize the high-resolution outputs. Chang *et al.* (2004 ▸) proposed a manifold learning technique as a replacement to the nearest neighbour strategy. Later, Yang *et al.* (2008 ▸, 2010*b* ▸) advanced this nearest neighbour correspondence to a more sophisticated sparse coding formulation. Other mapping techniques have also been used such as simple functions (Yang *et al.*, 2013 ▸), random forests (Schulter *et al.*, 2015 ▸), kernel regression (Kim & Kwon, 2010 ▸) and anchored neighbourhood regression (Timofte *et al.*, 2013 ▸, 2014 ▸), improving either the mapping accuracy or speed. Recently, Dong *et al.* (2014 ▸, 2016 ▸), inspired by the recent success of deep learning applications, explored its potential use in super-resolution approaches. In their proposed method, a convolutional neural network (CNN) was used as the mapping function between patches with low and high pixel count, as CNNs can learn how to map patches with a low pixel count to ones with a high pixel count, and optimize that learning in an end-to-end fashion. During experiments, their network achieved superior performance compared to the other state-of-the-art techniques, hinting that deep-learning-based super-resolution approaches may lead to future improved performance. Based on their research, new improved variations have been proposed (Kim *et al.*, 2015 ▸; Ledig *et al.*, 2016 ▸), with the main focus being better upscaling of natural images using deeper or more complex CNN architectures.

Inspired by this previous work, we propose here a super-resolution deep learning approach that aims to upscale the sinograms of X-ray tomograms (Section 2.2). Previous research has attempted sinogram upscaling. These methods range from methods based on linear interpolation (Brooks *et al.*, 1978 ▸), using a frequency consistency condition (Pohlmann *et al.*, 2014 ▸), using partial differential equations (Kostler *et al.*, 2006 ▸), using dictionary learning (Li *et al.*, 2014 ▸; Zhang & Sonke, 2013 ▸), methods based on directional interpolation (Zhang & Sonke, 2013 ▸; Bertram *et al.*, 2009 ▸) and methods using a combination of approaches (Kalke & Siltanen, 2014 ▸; Li *et al.*, 2012 ▸; Weiss *et al.*, 1982 ▸). However, we believe that with the recent progress of machine learning, and specifically of approaches utilizing fully convolutional networks, better solutions to this challenging ill-posed problem can be offered.

Our approach is primarily influenced by Dong *et al.*’s work (Dong *et al.*, 2014 ▸, 2016 ▸) as it offers a versatile structure compared with the recently published architectures that are primarily developed for the upscaling of *natural* images, in which the data can be more complex and less well constrained. Additionally, their network structure is simple and lightweight, which allows it to be quickly retrained for the upscaling of different classes of tomograms. However, since Dong *et al.*’s approach is designed for the upscaling of natural images, it is likely not suited to perform optimally on tomography data; this was confirmed in preliminary tests. For that reason it has not been used for comparisons in the later experiments; instead we would compare our approach with a popular directional interpolation technique proposed by Bertram *et al.* (2009 ▸) and cubic interpolation.

The main contribution of this paper is a new super-resolution deep learning approach, which achieves sinogram upscaling with better accuracy and faster execution times compared with known interpolation techniques. Additionally, as our approach uses a lightweight convolutional network, it allows sinogram upscaling of a wide variety of samples, as the network can easily and quickly be retrained to learn priors for new types of samples on a per experiment basis. This makes the approach practically useful, as in computed tomography experiments the samples under study can differ greatly and therefore being able to retrain the network relatively quickly is important.

This upscaling approach, then, provides the researchers with the ability to capture data at higher frequencies, which is significant during studies of temporal changes within the sample volume. During high-frequency capture, a time-series of tomograms with fewer projections is attained, due to the period of time required to take a higher number of them being prohibitive. Researchers can then selectively upscale the sinograms and therefore increase the number of projections computationally after capture has taken place. Furthermore, once our network is trained it can upscale each sinogram at a faster rate compared with other analytical methods, which is important as a tomogram time-series is typically comprised of multiple tomograms, each with potentially thousands of sinograms, and so are computationally expensive to reconstruct.

The remainder of this paper is organized as follows. Section 2 provides details about the challenge of the data itself, the nature of the tomograms, and background information on how they are reconstructed to voxel volumes. Section 3 presents our super-resolution deep learning approach, and provides details about the training procedure. Sections 4 presents the experiments that took place, examining the effects of parameters and application to real-world data. Finally, Section 5 concludes the paper.

## 2. Background on 4D datasets   

### 2.1. The nature of the datasets   

The datasets used later in the experiments of Section 4 are micro-computed tomographic datasets captured at Diamond Light Source’s I13-2 beamline (Bodey & Rau, 2017 ▸). For the capture of each tomogram, X-ray projections of the sample are captured from multiple angles within a sweep of 180°. This series of projections comprises a representation of the tomogram, that later using specialized software (Atwood *et al.*, 2015 ▸) is reconstructed into a 3D representation consisting of voxels. It is common practice for facilities such as Diamond Light Source to capture these tomograms with a low number of projections, allowing for greater time resolution of the experimental process that is being measured. These rapidly collected datasets are usually part of an even larger multidimensional (*e.g.* time series) dataset, which in total captures a whole temporal event, such as corrosion, deformation or failure of materials. The reduced number of projections in the tomograms, though, leads to a correspondingly low quality reconstruction of the voxel representation of the tomogram, which makes the clear identification and detection of their internal components exceedingly difficult. This can sometimes be addressed using iterative reconstruction methods such as SIRT (Trampert & Leveque, 1990 ▸), SART (Andersen & Kak, 1984 ▸) and CGLS (Zhu *et al.*, 1997 ▸), or more complex methods such as model building approaches, but these are often very computationally expensive and so not computationally plausible for application on large volumes of data, such as seen here.

### 2.2. Sinogram space   

**Figure 1:**
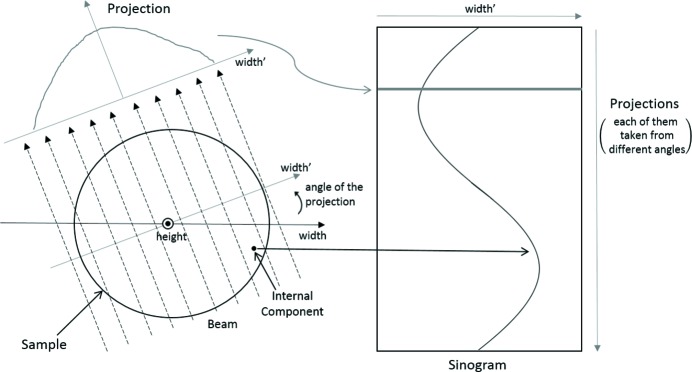
The formation of a sinogram. Internal components create sinusoidal lines in the sinogram as they rotate with the sample.

**Figure 2:**
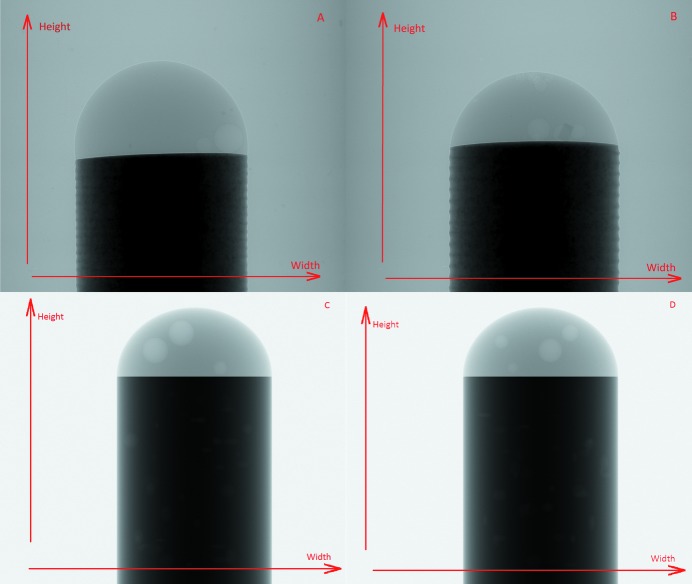
Images of projections (the 1801th projection out of 3601) from the different datasets used in this paper. (A) From the real-world data used for training. (B) From the real-world data used for testing. (C) From the synthetic data used for training. (D) From the synthetic data used for testing. The sample in the real-world data is an aluminium pin with magnesium deposits that reacts with the droplet of salt water on the top of the pin which produces hydrogen air bubbles. The synthetic data were constructed trying to simulate the real world counterpart.

**Figure 3:**
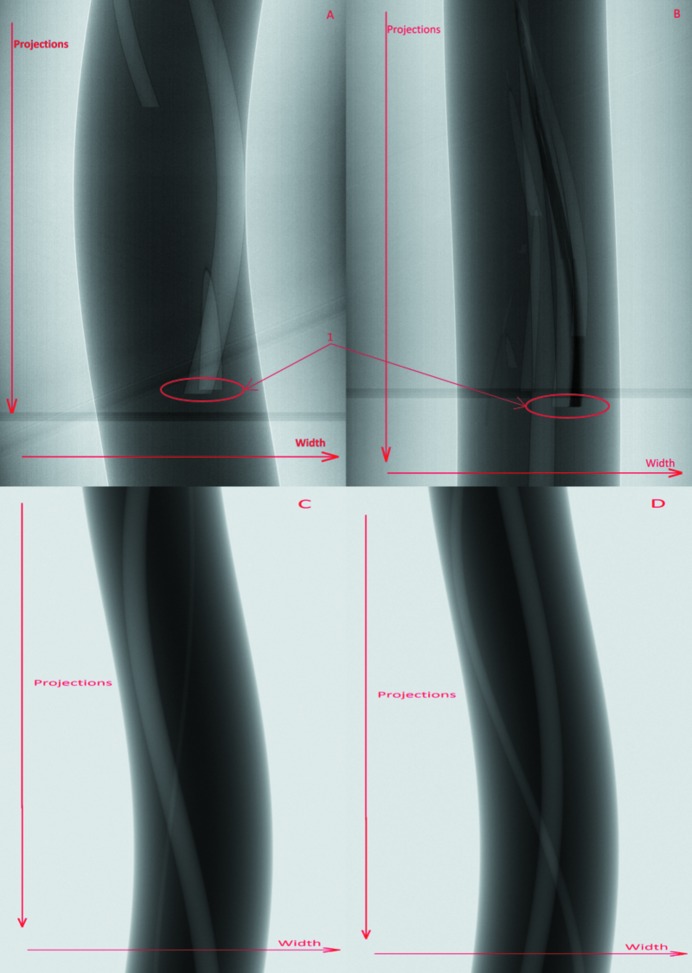
Images of sinograms (the 1001st pixel row counting from the top for the real-world data, the 201th pixel row for the synthetic data) from the different datasets used in this paper. (A) From the real-world data used for training. (B) From the real-world data used for testing. (C) From the synthetic data used for training. (D) From the synthetic data used for testing. These sinograms correspond to the height where hydrogen bubbles are expected to be found. As can be seen in the real-world data, the sinograms suddenly stop (marker 1) as the bubbles surface on the top of the sample and pop. This was purposely avoided in the synthetic data in order to check the accuracy of our method when the conditions are nominal. The image ratio is different from the original for presentation purposes.

In addition to the representation of a tomogram as a series of projections, it can also be represented as a series of sinograms. Each sinogram is created by stacking rows of pixels that correspond to a specific height in the sample from all the different projections, starting from the row captured first and ending with the row captured last (Figs. 1–3). For better understanding, let us denote the size of each projection as 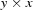, where *y* and *x* are the projection’s height and width, respectively. Also, let us denote the number of projections as θ, because this can also be thought of as the number of different angles from which projections have been taken. After the transition from the projection space to the sinogram space, there will be *y* sinograms in total, with each of them having size 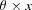. This representation receives its name from the sinusoidal lines present in the sinograms, a product of the trace that different internal components create as they are rotated with the sample (Fig. 1 ▸). From the above, we can suggest that tomograms with reduced number of projections θ can be expressed as *undersampled* tomograms. An effect of this is the presence of a reduced number of rows/angles in their sinograms. Following this point, we will refer to tomograms with such a reduced number of projections as being *undersampled*.

When studying high-speed processes, it is common before or after the acquisition of a series of high-speed, undersampled tomograms to capture a low-speed, highly sampled tomogram to help resolve details and understand the system as a whole. We will refer to these tomograms as *fully sampled* tomograms. Using these fully sampled datasets, it is possible then to learn important information that would allow the effective upscaling of the θ-axis in the sinograms of undersampled datasets. It is the aim of this work to use deep learning to achieve this by building and training a convolutional neural network using these fully sampled datasets, and applying the resultant model to the undersampled datasets in order to increase the number of their projections.

### 2.3. The datasets used in experiments   

In this work we are using both real-world and synthetic datasets. The real-world datasets were captured at Diamond Light Source to observe a corrosion process on a metal test sample. This was initiated by applying a drop of salt water to the top of a 500 µm-diameter aluminium pin with magnesium deposits within it (see example images in Figs. 2 ▸ and 3 ▸). The sample is mounted on the beamline sample stage, and the salt water drop is added to the sample, starting the corrosion process. As soon as this has started, a series of high-speed tomography data collections (91 projections per collection over 180°, using a fly scan or continuous rotation methodology) were acquired capturing the corrosion process as it occurs. Once the corrosion process has finished, or slowed to a stable level, and before the sample is removed, a final scan with a much higher number of projections (3601 projections, again as a fly or continuous scan) is collected. It is at this moment that the most representive highly sampled tomogram can be captured for this particular experiment, as different layers of the sample have undergone different levels of corrosion in our case. In principle, representive highly sampled tomograms could be captured at the beginning or at both the beginning and end of the experiment. It is then possible for our method to train using the sinograms of these tomograms and then upscale the component sinograms of the 4D data collection.

This high-quality scan is important for many purposes; in this case, it is highly representative of the time series of data, as no significant changes have occurred in the sample, which is needed for the method presented here. Originally, this high-quality scan was collected so that the result of the corrosion events under study could be easily identified manually, to guide the analysis in the undersampled data which contains more noise. Similarly, other 4D datasets in the Diamond Light Source are collected along with one or more highly sampled tomograms. For the real-world datasets used in our approach these tomograms are captured at the end of the sequence, as this is when they contain features that have evolved within the tomograms of the 4D datasets. However, it is a common strategy, depending on the experiment, to collect one or more highly sampled datasets at points where the resulting tomogram will well represent the 4D dataset tomograms partially or as a whole; this could be the beginning or end of a time series. This collection strategy is not performed solely to facilitate our approach, as it is already regularly performed to assist the researcher in the post-processing of the 4D datasets. This helps our approach to have a wider application, as one or more representative tomograms will most likely be available as part of standard protocols.

All the projections have a size of 2160 × 2560 pixels; it takes approximately 30 s for the capture of each of the undersampled tomograms in the time-series and 5 min for the final, fully sampled scan. The data are stored using the NeXuS data format (Könnecke *et al.*, 2015 ▸) which is built upon the HDF5 format (.h5) (The HDF Group, http://www.hdfgroup.org/HDF5/), which enables the researchers at Diamond to easily process the datasets and keep a detailed history as the data are processed through various steps (Atwood *et al.*, 2015 ▸). The real-world datasets (160 GB) are available on Zenodo (Bellos *et al.*, 2018*a* ▸). Apart from these real experimental data, it is also important to investigate our new analysis approach using synthetic data which can be generated in predictable ways. Therefore, synthetic data were created using the *TomoPhantom* toolbox (Kazantsev *et al.*, 2018 ▸). The data were created through the use of simple geometric objects like cylinders and ellipsoids in an effort to simulate their real life counterparts, broadly representing the real life datasets used here [see Figs. 2(C) and 2(D) ▸]. Because of current limitations of the *TomoPhantom* toolbox the size of the synthetic projections is 1080 × 1280 pixels, again having 3601 projections. The synthetic projections produced by *TomoPhantom* are simulated using a step-scan collection approach. Specifically, the pin is simulated via a cylinder primitive, and the droplet of salt water via an ellipsoid, cut in half. The hydrogen bubbles, a product of the corrosion in the real-world data, are simulated with randomly placed spheres of random radii (numbering three in the training synthetic dataset and four in the testing one). Additionally, the magnesium deposits in the pin are simulated with 40 randomly placed ellipsoids with random elliptic radii for each in both training and testing dataset. The noise present in the real-world data is simulated using a Poisson distribution. In the real-world data, however, temporal events occur during capture (the corrosion creates hydrogen bubbles). Such events produce abrupt changes in the sinogram [*e.g.* see marker 1 in Figs. 3(A) and 3(B) ▸]. This produces an extra challenge while upscaling, as analytical interpolation methods are not able to account for this step change, compared with our method that may be able to learn from the provided data. The software to generate the noisy datasets as well as the noiseless training and testing datasets themselves can be found online (Bellos *et al.*, 2018*b* ▸).

## 3. The Upscaling Deep Neural Network (UDNN)   

### 3.1. Network architecture   

We propose a convolutional network architecture designed to upscale the sinograms of the earlier mentioned tomograms with accuracy greater than previous analytical interpolation techniques. As described in Section 2, the θ-axis of each sinogram corresponds to different source projections, and, since we aim to increase artificially the number of projections, the proposed approach is designed to effectively upscale the sinograms along their θ-axis. Subsequently, this requires the estimation of the intermediate projections that are skipped during collection. Using such an upscaling approach, attaining tomogram reconstructions with better quality would become achievable using fewer acquisition steps, meaning that it would be possible to acquire data faster and also using less memory.

The network architecture is based on the work of Dong *et al.* (2016 ▸) and comprises three convolutional layers seperated by two rectified linear units (ReLUs) (Glorot *et al.*, 2011 ▸). The network layers jointly learn the underlying mapping between the input sinograms and the missing intermediate pixel-rows/projections. Because of that, the output of the network is the aggregation of these missing pixel-rows/projections. The desired upscaled sinogram is obtained by interlacing the pixel-rows/projections from the initial input sinogram with the output of the network (more details are given in Section 3.2). The three layers of the network are designed to perform the following three operations:

(1) *Feature extraction.* This operation generates a number of high-dimensional vectors for each pixel of the output using a local neighbourhood from the input sinogram. These vectors comprise the set of feature maps for the first layer, equal in number to the dimensionality of the vectors.

(2) *Introduction of non-linearity.* This operation maps the previous high-dimensional vectors to others with lower dimensionality via a non-linear function. This is desirable as it introduces sufficient non-linearity into the learned mapping, which is critical for the network’s overall performance.

(3) *Construction of the upscaled output.* This operation linearly combines the feature maps of the previous layer to synthesize the intermediate pixel-rows/projections, which interlaced with the input generate the desired sinogram with higher number of projections.

**Figure 4:**
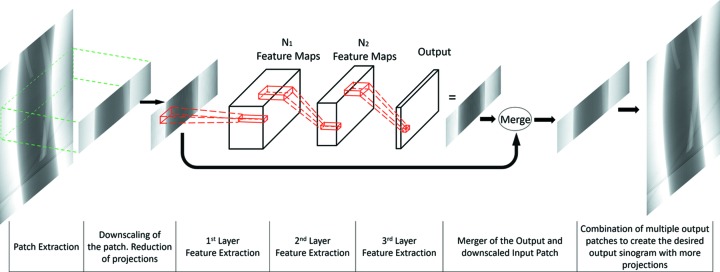
Graphical representation of the functionality of the proposed network. The downscaling of the sinograms is necessary during the network’s training with a set of fully sampled sinograms, but not for using the network after the training has finished.

In the following section we will present how these operations inspired the design of each convolutional layer. An overview of the complete network is depicted in Fig. 4 ▸.

### 3.2. Formulation   

**Figure 5:**
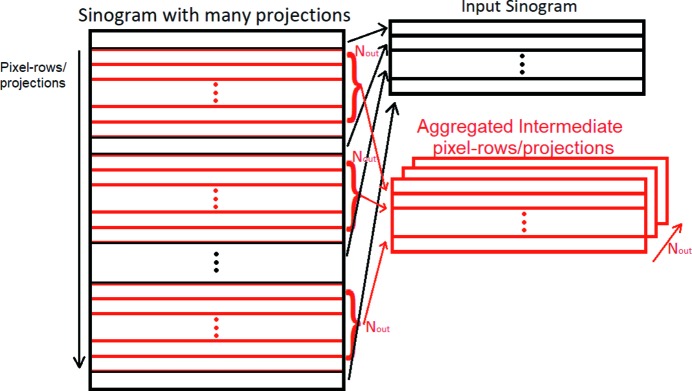
Diagram presenting how the input sinogram and the aggregation of the intermediate pixel-rows/projections are formed. Intermediate pixel-rows that are within two neighbouring pixel-rows/projections of the input sinogram are placed in different output channels.

Here we denote the input sinogram *I* and the desired learned mapping *F*. The goal is to recover from *I* an image 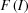 that would be as similar as possible to the ground truth image *O*. Here *O* is the aggregated intermediate pixel-rows/projections (see Fig. 5 ▸) extracted from a ground truth sinogram *G* of the fully sampled tomogram.

The first layer of the network can be expressed as 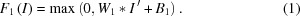
Here 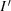 represents the input sinogram *I* appropriately padded with zeros, 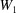 are the weights and 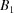 the biases of the first layer (identified by subscript 1). The symbol 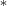 denotes the operation of convolution. In equation (1) the weights 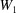 represent 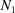 kernels of size 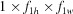, where 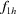 and 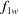 are the height and width of the kernels of this layer, respectively. In this first layer, 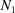 convolutions are applied on the input 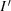 resulting in an output of 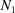 feature maps. Additionally, the biases are a 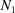-dimensional vector represented by 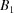. It is important to mention here that input sinograms are appropriately padded with zeros before the convolutions. The act of padding with zeros is common in CNNs as it is used to dictate the size of the output. In this layer the padding is used so each of its feature maps have the same size as *O*. Lastly, the function of ReLU is represented as a max function with the second argument being zero. ReLU’s functionality is to replace all the negative elements in the feature maps with zero.

Next in the architecture is another convolutional layer which provides the necessary depth and non-linearity to the network. The mathematical expression for it is as follows,
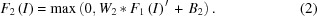
Here 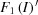 represents the feature maps 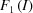 appropriately padded with zeros, so that the input and output feature maps have the same size. Also in this second layer (identified by subscript 2), 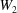 corresponds to 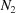 kernels of size 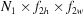, where 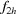 and 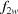 are the height and width of the kernels of this layer, respectively. The biases for this layer are represented by the 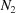-dimensional vector 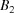. In this layer the 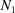 previous vectors are replaced with 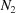 new ones, replacing also consecutively the corresponding feature maps. This is achieved by applying 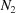 convolutions with kernels of 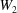.

At this stage, it be should noted that it is possible to add additional layers to deepen the network further at the expense of more training time. However, this was explored by Dong *et al.* (2016 ▸) and shown to have diminishing returns. For that reason, and since our proposed network is designed to upscale only greyscale intensity images in only one direction (along the *y*-axis; versus 2D colour upscaling), and it is important here to have short training times, the idea of adding additional layers was considered unnecessary.

Finally, the last layer constructs the output 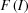 using the feature maps of the previous layer. The layer can be expressed as follows,
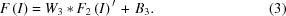
Here 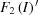 represents the feature maps 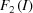 appropriately padded with zeros, so that 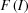 has the same size as *O*. Additionally, in the third layer (identified by subscript 3), 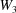 contains 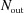 kernels of size 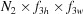, where 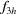 and 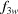 are the height and width of the kernel, respectively. 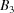 are the biases for this layer and are represented in an 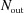-dimensional vector. In this layer the 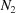 feature maps are being condensed using 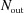 convolutions to the desired output. As we will explain in Section 3.3.3, 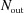 is the number of intermediate pixel-rows/projections between two neighbouring pixel-rows/projections of the input. Therefore, 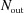 equals the upscaling factor minus one and by increasing it the network can be set to upscale the sinogram further. The parameters for this layer must be precisely fine-tuned as the estimation of each output pixel is the linear combination of multiple feature vectors, thus making it very error sensitive. This sensitivity will play an important role during the design of the training.

### 3.3. Training   

#### 3.3.1. Loss function   

In order for the network to configure its weights and biases 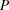 = 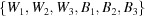, it has to train using already-known pairs of inputs and known outputs. This is achieved through the minimization of a loss function which calculates the error between the network’s predictions 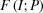 and the ground truth *G*. For this network the mean square error is being used as it is a good general loss function for estimating error; it is differentiable and values any inclination from ground truth equally. Given aggregated intermediate pixel-rows/projections *O* extracted from ground truth sinograms *G* of the fully sampled tomogram, and the available input sinogram *I*, the mean square error can expressed as
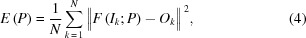
where *N* is the total number of pixels present in the aggregated intermediate pixel-rows/projections *O*.

#### 3.3.2. Backpropagation methods and learning rate   

The minimization of the loss function is typically achieved through backpropagation. In the experiments described later (Section 4), the adaptive momentum estimation (Adam) method (Kingma & Ba, 2014 ▸) is used to train the network. Adam has been shown to outperform stochastic gradient descent methods, like AdaGrad (Duchi *et al.*, 2011 ▸) and RMSProp (Tieleman & Hinton, 2012 ▸). There are two learning rates, one for the first two layers which starts from 0.01 and one for the last layer which starts from 0.001. Both decrease gradually as the network converges to the desired mapping. Two learning rates are used because of the sensitivity that the last layer demonstrates, as described earlier. The other hyperparameters described in the Adam algorithm are left at their default values (Kingma & Ba, 2014 ▸).

#### 3.3.3. Training and testing with our data   

As is described in Section 2.3, two datasets are reserved for training/validation (one from the synthetic and one from the real-world data) while the other two for testing (again from both real-world and synthetic data). The sinograms of datasets used for training/validation are split into two halves, one for training and the other for validation. Specifically from the training/validation dataset with real-world data, 1079 sinograms are reserved for training (2nd, 4th,…, 2158th) and 1079 for validation (3rd, 5th,…, 2159th), while from synthetic data 540 sinograms are reserved for training (1st, 3rd,…, 1079th) and 1079 (2nd, 4th,…, 1080th) for validation. The reason sinograms are selected in an alternating fashion for training and validation is because it splits them into two different, but also representative, halves. This is because the structure of the sample present in both real-world and synthetic data changes along its height axis, thus splitting the sinograms into two halves (one for the top part of the sample and one for the lower) would create two subsets that are not representative of the whole sample. It could be claimed that this might cause the training and validation sets to have a high correlation with each other and so generate misleading results; however, this was not observed in the later experiments when the accuracy of UDNN was tested in different datasets, respectively, for real-world and synthetic data.

In an effort to utilize the available sinograms during training as much as possible, the network does not use as inputs the whole sinograms but rather patches of them. This increases the number of training instances, which is important for sufficient training and also keeps the memory demands of the network low. The selection of the patches is executed as follows. In each sinogram, patches of 401 pixels in height and 2560 pixels in width are selected. From each of these patches, 11 pixel-rows/projections are used as the input for the network and another 10 as the ground truth. The pixel-rows/projections used as input are the 1st, 41st, 81st,…, 401st rows from the patch and the 21st, 61st,…, 381st are those used as ground truth. By doing that, the sinograms are being downscaled. This is because the sinograms of the fully sampled tomogram are comprised of 3601 projections, and the undersampled of only 91. This downscaling of the sinograms by a factor of 40 sets the angle difference between the pixel-rows/projections of the input and the ground truth similar to when they are part of an undersampled tomogram (Section 2). Also, since there is only one pixel-row/projection selected from the ground truth between each two neighbouring input pixel-rows/projections (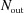 = 1), this sets the upscaling factor to 2×. This means that the output will be almost double in size along the *y*-axis (*almost*, because if the input has a length of 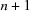 in the *y*-axis the output will have a length of 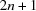). It is possible to increase the upscaling factor even more by adding more output channels (Fig. 5 ▸) where 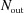 > 1 in the final convolutional layer. For example, if there were three output channels instead of one, the upscaling factor would be 4×. In this case each channel would try to predict different intermediate rows in the patch. The first channel would be for the 11th, 51st,…, 371st rows, the second for the 21st, 61st,…, 381st and the third for the 31st, 71st,…, 391st. In the following experiments we shall mostly test our network using an upscaling factor of 2×, but will demonstrate an upscaling factor of 4× in the real-world data.

Each sinogram patch is selected with a stride of 1, resulting in 3200 patches per sinogram. Through the use of a stride smaller than 401 (in this case 1), the patches will overlap with each other. This was chosen in order to diversify the input data as if the same sample was captured under different starting positions, utilizing therefore all 3601 different projections.

During the training of the network the sequence of input patches used for training are chosen each time from a different sinogram of those available. A sinogram will be ‘visited’ again to select and use one of its patches only after all other sinograms have been ‘visited’ and one of their patches has been used. The use of a patch from all the available training sinograms marks one ‘iteration’. The sequence of sinograms from which patches are drawn in each ‘iteration’ is selected randomly for each ‘iteration’. Additionally, the patches are provided in minibatches of ten for each backpropagation.

Based on the above, the number of patches reserved for training is 3452800 and 1728000 for the real-world and synthetic data, respectively, while also the same numbers of patches are reserved for validation. Obviously, using all the patches available for validation would result in an enormous time being needed for training. For this reason ten random patches are selected from each sinogram resulting in 10790 and 5400 patches used for validation, for the real-world and synthetic data, respectively. The validation is selected to occur after every 16 of the earlier-mentioned ‘iterations’ have been passed, meaning that each validation occurs after every 17280 and 8640 backpropagation iterations, again for the real-world and synthetic data, respectively. After every validation, a snapshot of the network parameters is captured and, depending on the trend of the error measured by the loss function, the learning rates are appropriately decreased (by a factor of ten) to enable the network to converge.

**Figure 6:**
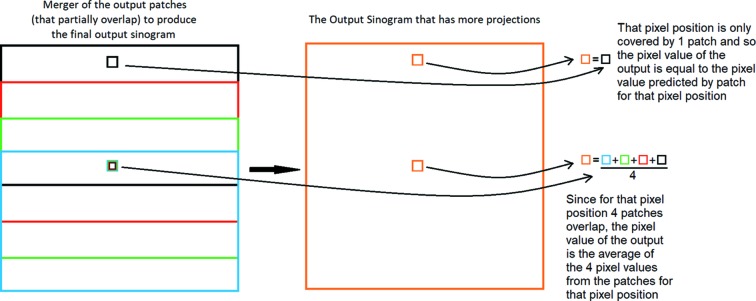
Diagram presenting how the output patches are merged to produce the output sinogram. At the end of the process each pixel of the output sinogram will be the average value of the pixels from the patches that overlap in each corresponding pixel-position.

The testing of the network was conducted on completely separate, different datasets, both with real-world and synthetic data to ensure the validity of the results. From the testing datasets 10% of the sinograms are downscaled by a factor of 40 (using 91 projection out of the 3601) and then upscaled with the trained network. These are the sinograms 1, 11, 21,… for the syntetic data and 2, 12, 22,… for the real (the first of the sinogram of the real-world datasets contains metadata making it therefore unusable). Since the network learns to upscale sinogram patches, the final upscaled sinogram is constructed through merging the output patches. This is applied by setting the value of each pixel of the output sinogram as the average value of the pixels from the different patches that overlap in the corresponding pixel-position (Fig. 6 ▸). Finally, it was shown in early experiments that the network performs better when the pixels in each real-world data patch are normalized. Therefore, each real-world data input is normalized before it is fed into the network and then the output is accordingly denormalized before it is compared with the ground truth in the evaluation stage. This process was not needed for the synthetic datasets because they are already normalized when generated.

#### 3.3.4. The UDNN model   

For the basic model of the network, UDNN (Upscaling Deep Neural Network), the first layer has a kernel size of 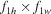 = 10 × 17, with 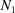 = 64 feature maps as well as padding (4, 8) accordingly for height and width. The second layer has a kernel size of 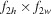 = 7 × 13, 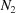 = 32 feature maps and (3, 6) for height and width padding. Finally, the last layer has kernel size of 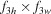 = 7 × 13, 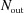 = 1 output channels (for an upscaling factor of 2×), and (3, 6) for padding. The number of features for the UDNN model are set based on the network proposed by Dong *et al.* (2016 ▸); however, the sizes of the kernels are changed here. They were selected in an effort to be adequately large, so that the network can accurately upscale the sinograms, but not excessively so, as that would affect the training and execution speed. Additionally, the padding was selected appropriately so that the output has the desired size (Section 3.2). It can be conjectured that further performance might be gained by exploring different feature numbers, sizes of kernels or training strategies. Based on that idea, we performed experiments exploring the two basic hyperparameters (number of feature maps and kernel sizes) and observed their effects on the overall performance. This is explored in Section 4 below.

## 4. Experiments   

### 4.1. Experiments with different hyperparameters   

In these experiments, we want to examine the accuracy of the UDNN model (Section 3.3.4) for different hyperparameter values. For these experiments we used real-world data with the setup described in Section 3.3.3. Bertram’s directional interpolation (Bertram *et al.*, 2009 ▸) is not used as a comparison as it is computationally expensive and the aim of these experiments is to compare the accuracy of different versions of the network (with different hyperparameters). Instead, cubic interpolation is used in order to provide a comparative baseline. Therefore, only training and validation was performed. Namely, we examine:

(1) The effects of the number of features on accuracy, where we examine whether a larger or smaller number of features in the hidden layers can improve accuracy.

(2) The effects of the sizes of kernels on accuracy, similarly to examine whether larger or smaller kernels can improve overall accuracy.

The reason we choose to experiment with these particular hyperparameters is because they comprise the fundamental hyperparameters affecting CNN architecture and training. It can be speculated that additional performance might be gained by experimenting with other hyperparameters (*e.g.* other number of features, kernel sizes, learning rates) or network architectures, but these questions are left for future research.

As a quality metric for the experiments we use the PSNR (peak signal-to-noise ratio). The PSNR is a widely used metric for evaluating image restoration, as high PSNR values signify higher restoration qualities. Additionally, we also use the SSIM (structural similarity index) metric (Wang *et al.*, 2004 ▸) to compare the structural similarity of the different reconstructions with a high-quality reconstruction (using 3601 projections) that acts as the ground truth. Both metrics are very commonly used to quantitatively measure the visual quality of an imaging system (image or volume) compared with another that is held as the ground truth in terms of quality.

All experiments were executed using a workstation with two 12 GB NVIDIA Titan Xp GPU, Intel Xeon E5-2620 v4 2.10 GHz CPU, 64 GB of RAM, using the Pytorch package (Ketkar, 2017 ▸) (version 0.4) for Python 3.6.4 and executing the training, validation and testing on GPU using CUDA 8.0 through Pytorch’s appropriate library.

#### 4.1.1. Effects of the number of features on performance   

One important parameter when it comes to designing a neural network is the number of features in each layer. In the UDNN model the number of features selected for the first layer is 64 and for the second 32. For this experiment the number of features of the UDNN is doubled to 128 and 64 (UDNN-128) and halved to 32 and 16 (UDNN-32), respectively, for the first two layers.

**Figure 7:**
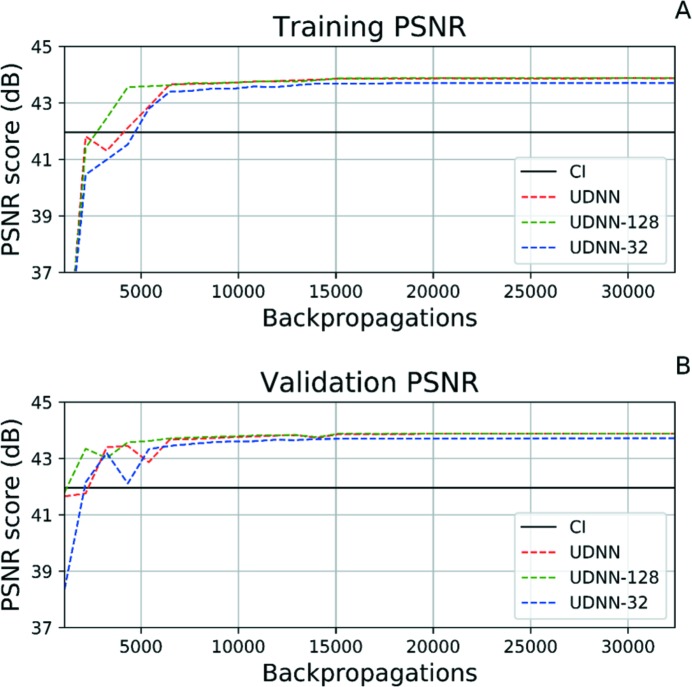
The training (A) and validation (B) PSNR graphs for the cubic interpolation (CI), the UDNN, the UDNN using 128–64 feature maps (UDNN-128) and the one using 32–16 (UDNN-32). The graphs depict the PSNR score from the initial 32400 backpropagations; after this point the network has converged and the PSNR score plateaus.

The PSNR score at the 32400th backpropagation is 43.88 dB for the UDNN, 43.89 dB for the UDNN-128 and 43.71 dB for UDNN-32. Based on these numbers and the dynamic response in Figs. 7(A) and 7(B) ▸, we can conclude that the addition of more feature maps helps the network to converge in a higher PSNR value, but with diminishing returns. Because of that, in the later experiments we use the UDNN-128 and UDNN model, but do not use or test a model with an even higher number of feature maps. Additionally, the UDNN-32 model with the fewer number of feature maps converged with an accuracy lower than the UDNN model. Therefore, we also can conclude that using less that 64–32 feature maps would not lead to satisfactory results.

### 4.2. Effects of the sizes of kernels on performance   

**Figure 8:**
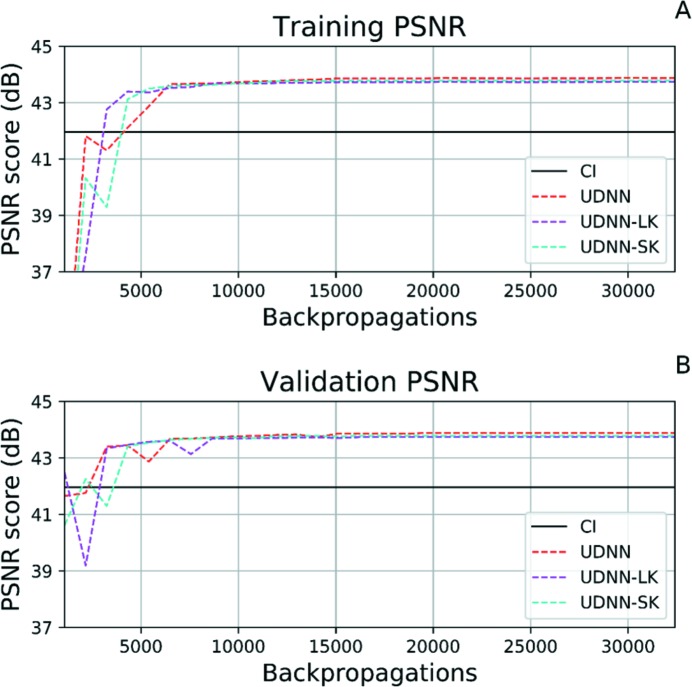
The training (A) and validation (B) PSNR graphs for the cubic interpolation (CI), the UDNN, the UDNN using large kernel sizes (UDNN-LK) and the one with small (UDNN-SK). The graphs depict the PSNR score from the initial 32400 epochs. The graphs do not show any further backpropagations as the network has converged and the PSNR score plateaus.

Next, the effect of size of the kernels in each layer was investigated. For the base UDNN model the kernels have size 10 × 17, 7 × 13, 7 × 13. For this experiment, two variations of the model are tested. One with larger sizes of kernels (UDNN-LK), specifically 12 × 19, 9 × 15, 9 × 15, respectively, for each of the three layers; and one where the kernels are smaller (UDNN-SK), specifically 8 × 15, 5 × 11, 5 × 11, respectively, for each of the three layers. The kernel sizes were increased by adding or subtracting two columns and rows from those UDNN in order to examine how the network’s behaviour changes with larger or smaller kernels generally in all layers. Results are shown in Fig. 8 ▸.

The PSNR score at the 32400th backpropagation is 43.88 dB for the UDNN, 43.74 dB for the UDNN-LK and 43.78 dB for the UDNN-SK. Based on these results and Figs. 8(A) and 8(B) ▸ we can infer that the enlargement or shrinkage of the sizes of the kernels does not offer better results regarding accuracy in practice. This is perhaps because with the increased kernel size the network takes into consideration pixel values from further away pixels which do not contain valuable information for the task of upscaling the central pixel. On the other hand, smaller kernels perhaps miss information which is occasionally crucial for upscaling of the central pixel, especially in our case where the angle difference between neighbouring rows/projections of the input is large (in only 91 projections the sample is rotated 180°).

### 4.3. Experiments with synthetic data   

As previously described, we also performed experiments on synthetic data that were created using the *TomoPhantom* toolbox (Kazantsev *et al.*, 2018 ▸). For these experiments we used the UDNN-128 model, as this is the best performing so far. Results are shown in Section 4.3.1.

#### 4.3.1. Synthetic data sinogram upscaling   

**Figure 9:**
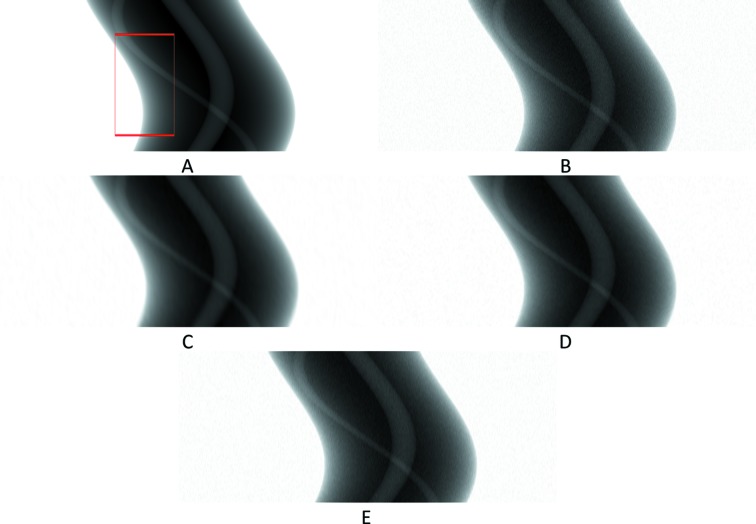
The 201st synthetic sinogram with only the intermediate row/projections (rows 21, 61,…, 3581, pixel-value [e^−1^:1]). (A) From the original dataset without noise. (B) From the original dataset with noise. (C) Prediction for these rows using UDNN-128 for upscaling. (D) Prediction for these rows using Bertram’s directional interpolation. (E) Prediction for these rows using cubic interpolation. Each image ratio is different from the original for presentation purposes. The red region in (A) is shown zoomed in Fig. 10 ▸.

Visual results can be seen in Figs. 9 ▸ and 10 ▸, and numerical results in Table 1 ▸.

#### 4.3.2. Synthetic data reconstructions   

**Figure 11:**
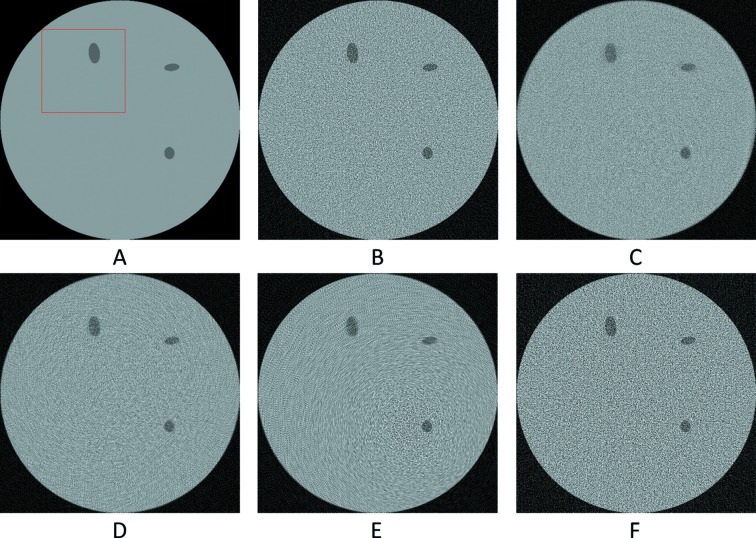
The reconstruction of the 601th synthetic sinogram (area [273:848, 273:848] of [1:1280,1:1280], pixel-value [0:0.5]). (A) From the original dataset without noise and 3601 projections. (B) From the original dataset with noise and 181 projections. (C) Using UDNN-128 for upscaling (181 projections). (D) Using the Bertram’s directional interpolation (181 projections). (E) Using cubic interpolation (181 projections). (F) From the original dataset with noise and 91 projections. Region of interest shown zoomed in Fig. 12 ▸.

The upscaled sinograms were later reconstructed by filter back projection using the Astra toolbox integrated into the *TomoPhantom* (Kazantsev *et al.*, 2018 ▸). Visual results can be seen in Figs. 11 ▸ and 12 ▸, and numerical results in Table 2 ▸.

### 4.4. Real-world data   

In addition to synthetic data we also tested our method on data provided by the Diamond Light Source [see Section 2.3 for details, Figs. 2(B) ▸ and 3(B) ▸]. For these experiments we used the UDNN-128 model.

#### 4.4.1. Experimental complexities   

For the upscaling of the sinograms in real-world data, there are two important issues which can arise.

The first is that the rotation axis between the different tomograms of the time-series may change. This is expressed in the sinograms collected at the Diamond Light Source as a shift of the collected data along the *x*-axis. However, due to the nature of CNNs this does not affect the final result; this is because the respective kernels within the convolutional layers are shifted to perform the operation of convolution, which makes the whole network translationally invariable.

The second issue is that the sample may be shifted perpendicular to the orientation of the detector. This effect can change the shape of the sinusoidal lines in the sinograms. While that could pose a problem for the correct upscaling of the sinograms, it was not observed during the experiments. As a matter of fact, the sample used in the real-world testing was in a different position compared with the training sample. We speculate that this behaviour is due to the light-weight structure of our network which helped it to not overtrain using the training dataset, leaving the door open for potential recycling of the trained network across varied capture regimes.

In any case though, commonly the researchers during the collection of 4D datasets strive to minimize the movement of the sample as it can become troublesome for many post-processing techniques.

#### 4.4.2. Real-world data sinogram upscaling   

**Figure 13:**
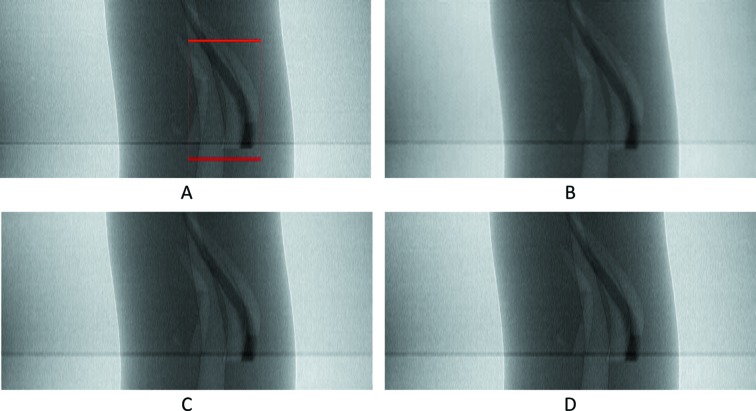
The 1002th real-world sinogram with only the intermediate row/projections (rows 21, 61,…, 3581, pixel-value [0.59:0.74]). (A) From the original dataset. (B) Prediction for these rows using UDNN-128 for upscaling. (C) Prediction for these rows using Bertram’s directional interpolation. (D) Prediction for these rows using cubic interpolation. Each image ratio is different from the original for presentation purposes. Region of interest is shown zoomed in Fig. 14 ▸.

Visual results can be seen in Figs. 13 ▸–16 ▸ ▸ ▸, and numerical results in Tables 3 ▸ and 4 ▸.

**Figure 15:**
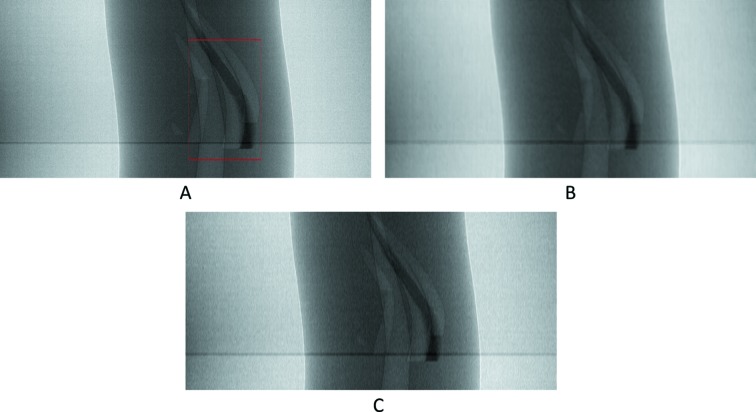
The 1002th real-world sinogram with only the intermediate row/projections (rows 11, 21, 31, 51, 61, 71,…, 3571, 3581, 3591, pixel-value [0.59:0.74]). (A) From the original dataset. (B) Prediction for these rows using UDNN-128 with three output channels. (C) Prediction for these rows using cubic interpolation. Region of interest is shown zoomed in Fig. 16 ▸.

Additionally, as explained in Sections 3.2 and 3.3.3, we also tested our network with a higher upscaling factor (of 4×), by adding additional output channels in the final convolutional layer in our network, 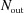 = 3. The reason we jump from two to four and not to three is because it is intuitively easier, as the input patch with 400 rows (+1 in the end) in height is divisible by 4. For this experiment we did not apply the directional interpolation method as it was not designed to be used iteratively. Results are shown in Figs. 15 ▸ and 16 ▸ and Table 4 ▸.

#### 4.4.3. Real-world data reconstructions   

**Figure 17:**
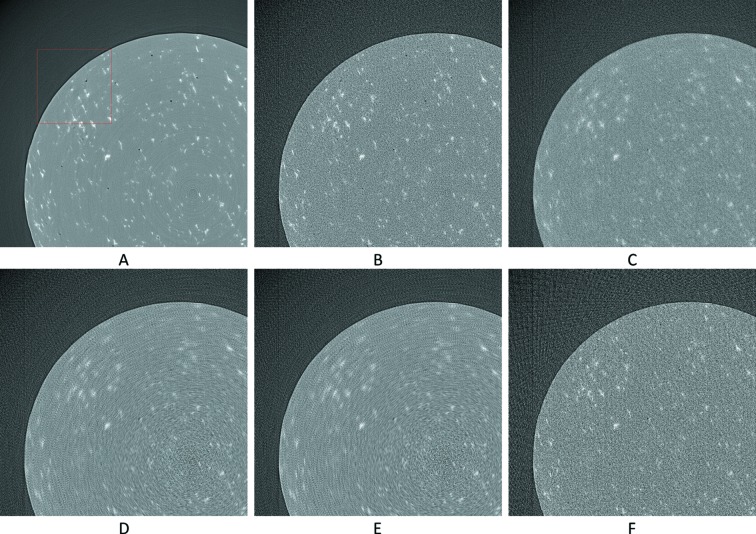
The reconstruction of the 1502th real-world sinogram (area [501:1500, 501:1500] of [1:2560,1:2560], pixel-value [−1/2560:1/2560]). (A) From the original dataset with 3601 projections. (B) From the original dataset with 181 projections. (C) Using UDNN-128 for upscaling (181 projections). (D) Using the Bertram’s directional interpolation (181 projections). (E) Using cubic interpolation (181 projections). (F) From the original dataset with 91 projections. Region of interest shown zoomed in Fig. 18 ▸.

The upscaled sinograms were later reconstructed by filter back projection using the Savu Framework (Wadeson & Basham, 2016 ▸) and the AstraReconGpu plug-in. For the calculation of SSIM for these data only the window [501:1500, 501:1500] was used. This was because the AstraReconGpu plug-in adds a circular mask of NaN elements, so these and some of the surrounding air were excluded from analysis. Visual results can be seen in Figs. 17 ▸–20 ▸ ▸ ▸, and numerical results in Tables 5 ▸ and 6 ▸.

**Figure 19:**
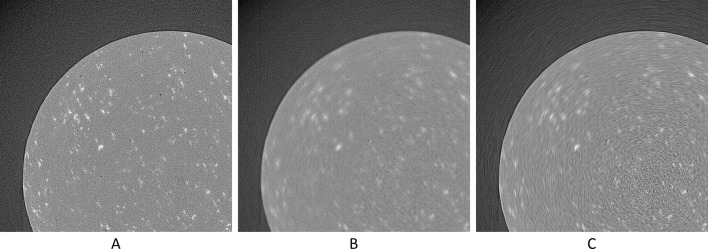
The reconstruction of the 1502th real-world sinogram (area [501:1500, 501:1500] of [1:2560,1:2560], pixel-value [−1/2560:1/2560]). (A) From the original dataset with 361 projections. (B) Using UDNN-128 and an upscaling factor of 4× (361 projections). (C) Using cubic interpolation (361 projections).

Apart from using an upscaling factor of 2× we also used an upscaling factor of 4× (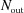 = 3). Results are shown in Figs. 19 ▸ and 20 ▸ and Table 6 ▸.

**Figure 21:**
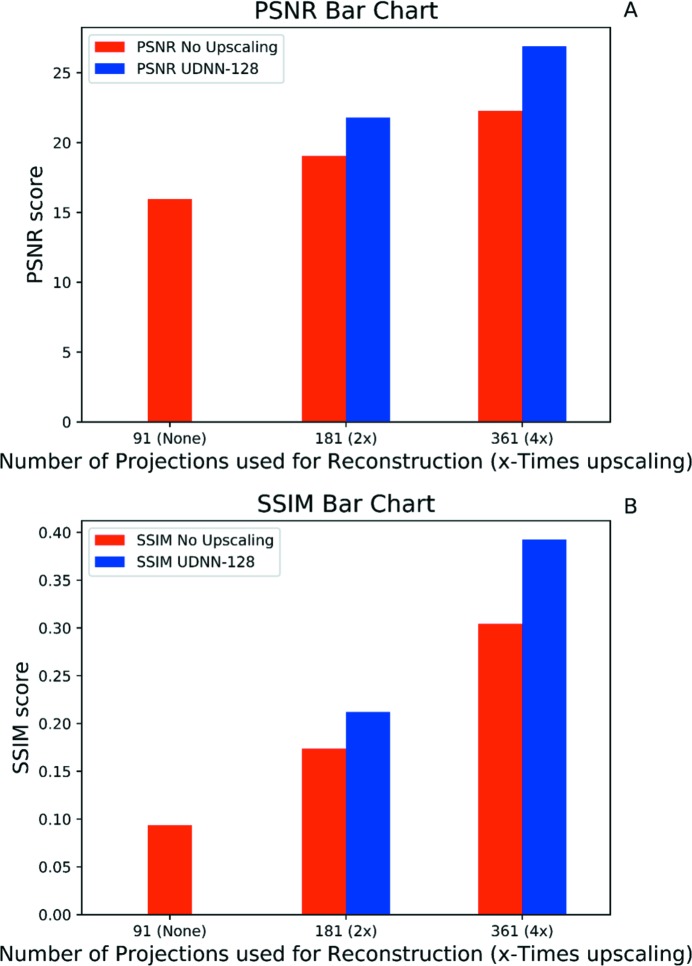
Bar charts showing the progression of the two used metrics (PSNR and SSIM), for the UDNN-128 network as the sinogram upscaling factor increases and therefore also the number of projections.

For comparison, Fig. 21 ▸ shows the previous numerical PSNR and SSIM scores presented as two bar charts. They depict how the scores change as the number of projections used for reconstruction is increased.

### 4.5. Discussion   

Based on the above results, we can observe that the UDNN-128 version of our network was able to slightly but consistently outperform the directional (Bertram *et al.*, 2009 ▸) and cubic interpolation in both PSNR and SSIM metrics (Tables 1 ▸–6 ▸). In addition, as can be seen in both synthetic and real-world data (Figs. 11 ▸–12 ▸, 17 ▸–20 ▸ ▸ ▸), the reconstructions after the use of our UDNN upscaling method contain fewer rotational artefacts, and have lower levels of noise. However, due to the loss of information in undersampled tomograms, it is difficult to restore detail especially in small components. This is a limitation for all upscaling techniques. This becomes more prevalent as the upscaling factor increases. For this reason, we consider that an upscaling factor larger than 4× will not be practically helpful despite probably having better relative performance in the metrics because of the noise reduction (Fig. 15 ▸). A potential solution to this issue would be the introduction of weights associated with these smaller internal components in the loss function during training; we will explore this in future research.

Apart from the improved restoration quality over other interpolation techniques, UDNN also offers fast execution times. For the upscaling of each sinogram, using UDNN-128 generally takes up to 1.7 s compared with 5.5 s of the directional method (Bertram *et al.*, 2009 ▸). Nonetheless, this is without considering the time required for training. For the UDDN-128 model, training essentially concludes after approximately 4 h, as the validation error later plateaus without reducing further. However, this is only required once per experiment. If we consider the case of a time-series with 20 tomograms each with 2160 sinograms it would take approximately 66 h for our workstation to upscale all sinograms using directional interpolation, but roughly 25 h with our method *including* the training session.

### 4.6. Example application to a time-series tomogram   

**Figure 22:**
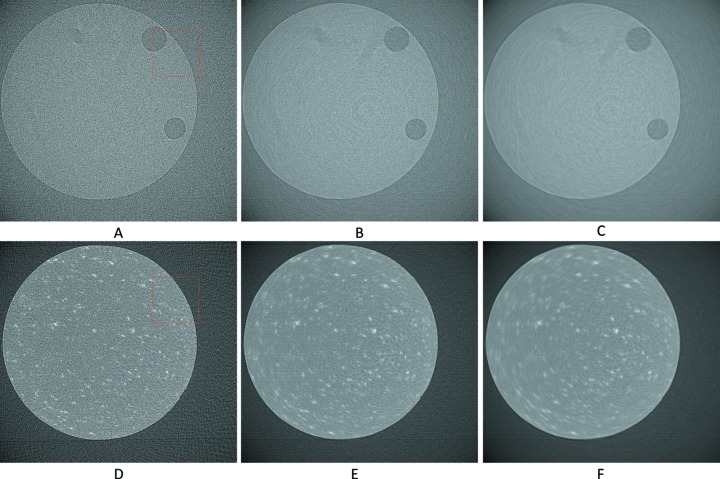
Applying our upscaling method on an undersampled tomogram that is part of a time-series. (A, B, C) Reconstruction of the 1101th sinogram (area [550:2000, 550:2000] of [1:2560,1:2560], pixel-value [−4.501 × 10^−4^:1.097 × 10^−4^]). (D, E, F) Reconstruction of the 1601th sinogram (area [550:2000, 550:2000] of [1:2560,1:2560], pixel-value [−1/2560:1/2560]). (A, D) From the original dataset with 91 projections. (B, E) Using UDNN-128 for upscaling resulting to 181 projections. (C, F) Using UDNN-128 for upscaling resulting to 361 projections. Region of interest presented zoomed in Fig. 23 ▸.

In Figs. 22 ▸ and 23 ▸ we present some reconstruction images, where our UDNN method was applied on an actual undersampled tomogram which is part of a time-series. Conveniently, this time-series has as its final, fully sampled tomogram the dataset used for training during experiments with real-world data, so retraining is not necessary here. The following reconstuctions are presented in order to visually demonstrate that our method does not behave differently when it is used to upscale tomograms collected with a low number of projections, as opposed to artificially created datasets where a few projections are selected from a complete dataset.

## 5. Conclusion   

To conclude, this paper presents a novel approach of upscaling sinograms along the θ-axis in tomograms that are part of 4D data collections using a super-resolution approach based on deep learning. The proposed approach, UDNN, using a convolutional neural network architecture and a fully sampled representative tomogram learns an end-to-end mapping between sinograms with low and high number of projections. Because of the prior provided by the fully sampled dataset, the network is able to train effectively and eventually surpass common interpolation techniques in accuracy. This in turn also leads to better improvement of the quality of the tomograms’ reconstructions, with fewer artefacts and less noise, enabling easier procesing of the tomograms for further analysis.

Based on the above experiments, the best performing version of UDNN, UDNN-128, was able to achieve better accuracy than Bertram *et al.*’s directional interpolation method and cubic interpolation (Bertram *et al.*, 2009 ▸).

We conjecture that additional accuracy or faster training/execution times can be further achieved by exploring different network architectures and/or training strategies. Beside that, one could also investigate network architectures for different upscaling factors or type of data (*e.g.* MRIs).

For the software used for our method see Bellos *et al.* (2018*c* ▸), and that for the datasets see Bellos *et al.* (2018*a* ▸,*b*) ▸.

## Figures and Tables

**Figure 10:**
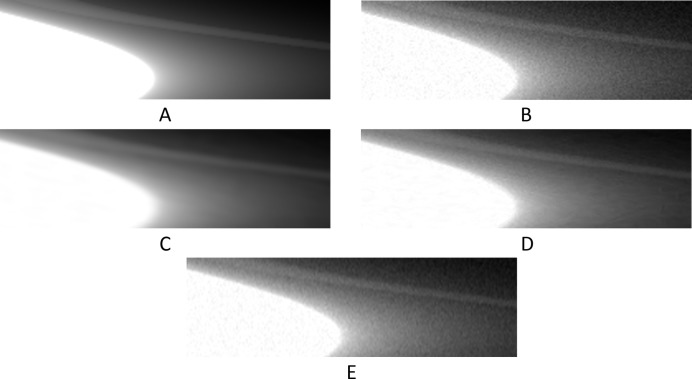
The selected area marked by the red window in Fig. 9(A) ▸ enlarged (width window [401:600] out of [1:1280] and for rows 821, 861,…, 3221, pixel-value [e^−1^: 1]). (A) From the original dataset without noise. (B) From the original dataset with noise. (C) Prediction for these rows using UDNN-128 for upscaling. (D) Prediction for these rows using Bertram’s directional interpolation. (E) Prediction for these rows using cubic interpolation. Note the clarity of the proposed method [panel (C)]. Each image ratio is different from the original for presentation purposes.

**Figure 12:**
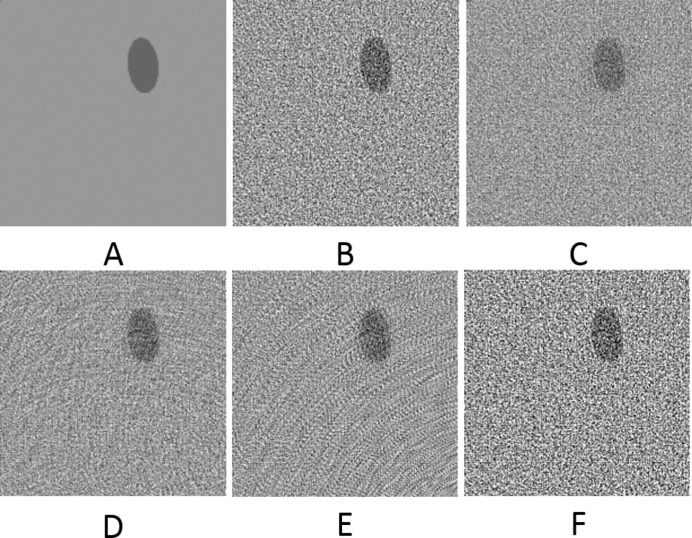
The selected area marked by the red window in Fig. 11(A) ▸ enlarged (area [373:573, 343:543] of [1:1280,1:1280], pixel-value [0:0.5]). (A) From the original dataset without noise and 3601 projections. (B) From the original dataset with noise and 181 projections. (C) Using UDNN-128 for upscaling (181 projections). (D) Using the Bertram’s directional interpolation (181 projections). (E) Using cubic interpolation (181 projections). (F) From the original dataset with noise and 91 projections. Note the with the proposed method (panel C) the presence of noise is decreased and that there are fewer rotational artefacts.

**Figure 14:**
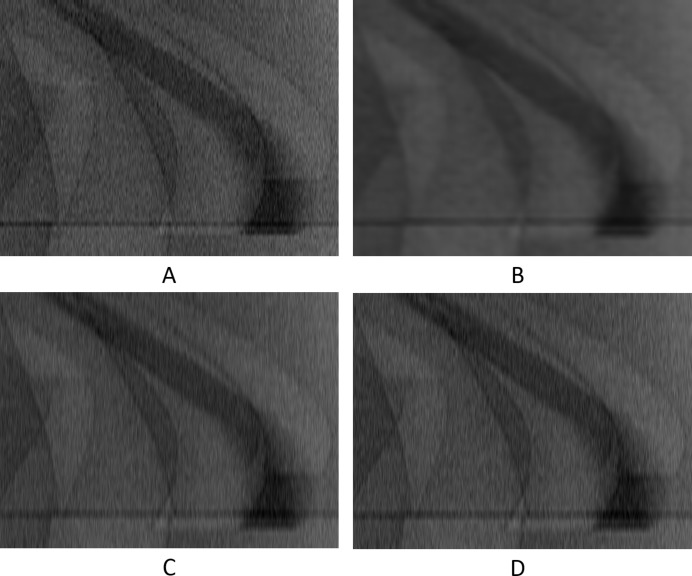
The selected area marked by the red window in Fig. 13(A) ▸ enlarged (width window [1301:1800] out of [1:2560] and for rows 821, 861,…, 3221, pixel-value [0.59:0.74]). (A) From the original dataset. (B) Prediction for these rows using UDNN-128 for upscaling. (C) Prediction for these rows using Bertram’s directional interpolation. (D) Prediction for these rows using cubic interpolation similar to Fig. 13 ▸. Similar to Fig. 10 ▸, with the proposed method (panel B) there is less noise introduced into the sinograms. Each image ratio is different from the original for presentation purposes.

**Figure 16:**
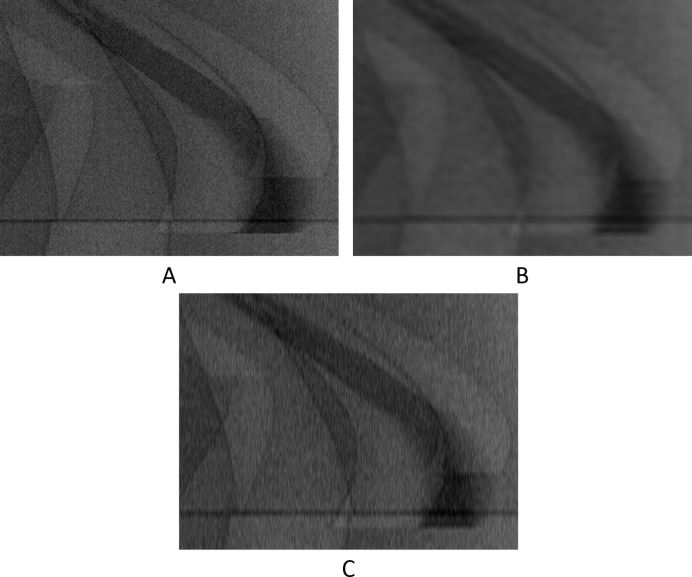
The selected area marked by the red window in Fig. 15(A) ▸ enlarged (width window [1301:1800] out of [1:2560] and for rows 811, 821, 831, 851,…, 3211, 3221, 3231, pixel-value [0.59:0.74]). (A) From the original dataset. (B) Prediction for these rows using UDNN-128 with three output channels. (C) Prediction for these rows using cubic interpolation. Again with the proposed method (panel B), there is less noise introduced into the sinograms. Each image ratio is different from the original for presentation purposes.

**Figure 18:**
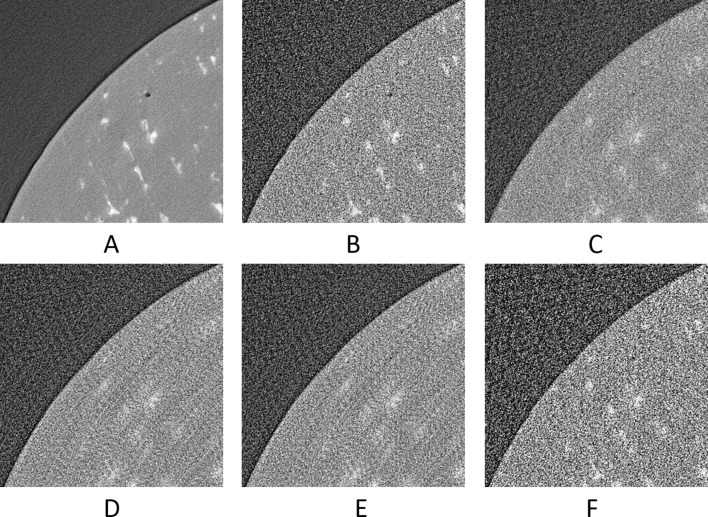
The selected area marked by the red window in Fig. 17(A) ▸ enlarged (area [651:951, 701:1001] of [1:2560,1:2560], pixel-value [−1/2560:1/2560]). (A) From the original dataset with 3601 projections. (B) From the original dataset with 181 projections. (C) Using UDNN-128 for upscaling (181 projections). (D) Using the Bertram’s directional interpolation (181 projections). (E) Using cubic interpolation (181 projections). (F) From the original dataset with 91 projections. Similar to Fig. 9 ▸, in the proposed method (panel C) the level of noise is lower compared with the other methods and there are fewer rotational artefacts.

**Figure 20:**
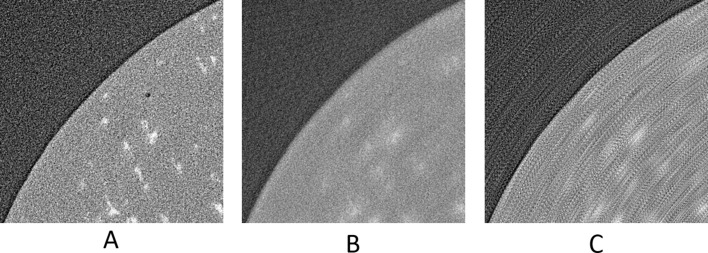
The selected area marked by the red window in Fig. 17(A) ▸ enlarged for Fig. 19 ▸ (area [651:951, 701:1001] of [1:2560,1:2560], pixel-value [−1/2560:1/2560]). (A) From the original dataset with 361 projections. (B) Using UDNN-128 and an upscaling factor of 4× (361 projections). (C) Using cubic interpolation (361 projections). Again, the proposed method (panel B) introduces less noise and fewer rotation artefacts.

**Figure 23:**
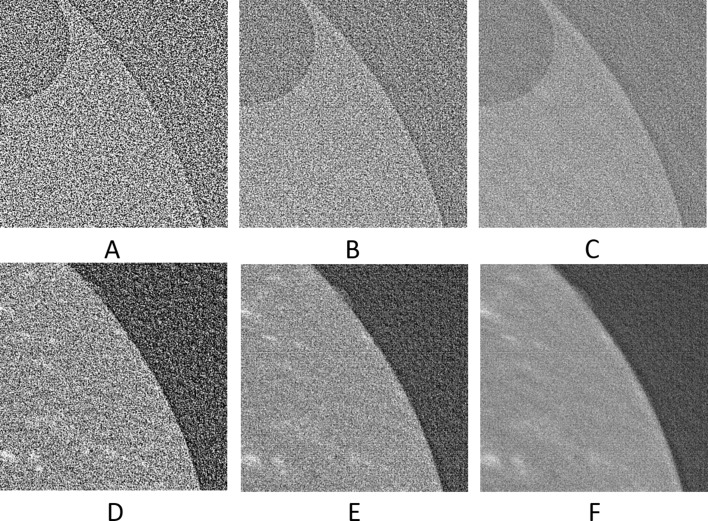
The selected area marked by the red window in Fig. 22(A) ▸ enlarged (area [1501:1800, 701:1000] of [1:2560,1:2560]). (A, B, C) Reconstruction of the 1101th sinogram (area [550:2000, 550:2000] of [1:2560,1:2560], pixel-value [−4.501 × 10^−4^:1.097 × 10^−4^]). (D, E, F) Reconstruction of the 1601th sinogram (area [550:2000, 550:2000] of [1:2560,1:2560], pixel-value [−1/2560:1/2560]). (A, D) From the original dataset with 91 projections. (B, E) Using UDNN-128 for upscaling resulting in 181 projections. (C, F) Using UDNN-128 for upscaling resulting in 361 projections. Similar to the earlier Figs. 12 ▸, 18 ▸ and 20 ▸, the proposed method (panels B, C, E and F) produces more clear reconstructions with less noise.

**Table 1:** PSNR score for upscaling the sinograms 1, 11,…, 108 of the synthetic dataset used for testing [Section 2.3, Figs. 2(D) ▸ and 3(D) ▸] For the calculations, the intermediate row/projections (rows 21, 61,…, 3581) of the original sinograms without noise were used as ground truth. Noisy Proj is the PSNR from the error between the downscaled noisy sinograms (sinograms 1, 11,…, 108 and for rows 21, 61,…, 3581) and the downscaled noiseless sinograms (again sinograms 1, 11,…, 108 and rows 21, 61,…, 3581). For the other columns the sinograms (1, 11,…, 108) were downscaled to a height of 91 (or 91 projections) and then upscaled using the different methods to 181 (2× upscaling), therefore creating the predictions for the rows 21, 61,…, 3581 of the original (intermediate row/projections). The PSNR for these predictions was then calculated against the downscaled noiseless sinograms. In the table below CI stands for cubic interpolation and DI for directional interpolation. Best result shown in bold.

	Noisy Proj	CI	UDNN-128	DI
Testing PSNR	40.88 dB	42.53 dB	**48.43** dB	48.15 dB

**Table 2:** PSNR and SSIM score of the reconstructions using the sinograms 1, 11,…, 108 of the synthetic dataset used for testing [Section 2.3, Figs. 2(D) ▸ and 3(D) ▸] For the calculations, a reconstruction of the original sinograms without noise (3601 projections) was used as ground truth. The 181 and 91 Noisy Proj correspond to reconstructions made from sinograms that were only downscaled accordingly from the original. The other columns correspond to reconstructions made by these sinograms, where they are initially downscaled to a height of 91 (or 91 projections) and then upscaled to 181 (2× upscaling) with the available methods. In the table below CI stands for cubic interpolation and DI for directional interpolation. Best results for each metric highlighted in bold.

	181 Noisy Proj	CI	UDNN-128	DI	91 Noisy Proj
Testing PSNR	14.82 dB	15.67 dB	**17.67** dB	17.40 dB	11.67 dB
Testing SSIM	0.0391	0.0454	**0.0625**	0.0607	0.0211

**Table 3:** PSNR score for upscaling the sinograms 2, 12,…, 217 of the real-world dataset used for testing [Section 2.3, Figs. 2(B) ▸ and 3(B) ▸] For the calculations, the intermediate row/projections of the original sinograms were used as ground truth. Namely, the sinograms (2, 12,…, 217) were downscaled to a height of 91 (or 91 projections) and then upscaled using the available methods to 181 (2× upscaling), therefore creating the predictions for the rows 21, 61,…, 3581 of the original (intermediate row/projections). The PSNR for these predictions was then calculated against the downscaled noiseless sinograms. In the table below CI stands for cubic interpolation. Best result shown in bold.

	CI	UDNN-128	DI
Testing PSNR	42.20 dB	**43.90** dB	42.59 dB

**Table 4:** PSNR score for upscaling the sinograms 2, 12,…, 217 of the real-world dataset used for testing [Section 2.3, Figs. 2(B) ▸ and 3(B) ▸] For the calculations, the intermediate row/projections of the original sinograms were used as ground truth. Namely, the sinograms (2, 12,…, 217) were downscaled to a height of 91 (or 91 projections) and then upscaled using the available methods to 361 (4× upscaling), therefore creating the predictions for the rows 21, 61,…, 3581 of the original (intermediate row/projections). The PSNR for these predictions was then calculated against the downscaled noiseless sinograms. In the table below CI stands for cubic interpolation. Best result shown in bold.

	CI	UDNN-128
Testing PSNR	42.01 dB	**43.89** dB

**Table 5:** PSNR score for reconstruction of the upscaled sinograms 2, 12,…, 217 of the dataset with real-world data used for testing [Section 2.3, Figs. 2(B) ▸ and 3(B) ▸] For the calculations, a reconstruction of the original sinograms (3601 projections) was used as ground truth. The 181 and 91 Noisy Proj correspond to reconstructions made from sinograms that were only downscaled accordingly from the original. The other columns correspond to reconstructions made by these sinograms, where they are initially downscaled to a height of 91 (or 91 projections) and then upscaled to 181 (2× upscaling) with the available methods. In the table below CI stands for cubic interpolation and DI for directional interpolation. Best result for each metric highlighted in bold.

	181 Proj	CI	UDNN-128	DI	91 Proj
Testing PSNR	19.03 dB	19.69 dB	**21.79** dB	20.13 dB	15.94 dB
Testing SSIM	0.1737	0.1504	**0.2122**	0.1627	0.0936

**Table 6:** PSNR score for reconstruction of the upscaled sinograms 2, 12,…, 217 of the dataset with real-world data used for testing [Section 2.3, Figs. 2(B) ▸ and 3(B) ▸] For the calculations, a reconstruction of the original sinograms (3601 projections) was used as ground truth. The 361 Noisy Proj correspond to reconstructions made from sinograms that were only downscaled to 361 projections from the original. The other columns correspond to reconstructions made by these sinograms, where they are initially downscaled to a height of 91 (or 91 projections) and then upscaled to 361 (4× upscaling) with the available methods. In the table below CI stands for cubic interpolation. Best result for each metric highlighted in bold.

	361 Proj	CI	UDNN-128 (*N*_out_ = 3)
Testing PSNR	22.28 dB	25.59 dB	**26.89** dB
Testing SSIM	0.3043	0.2163	**0.3925**
